# Mediastinal aberrant goiter characterized by high radiodensity on non-enhanced computed tomography: a case report

**DOI:** 10.1186/s44215-022-00004-3

**Published:** 2022-09-27

**Authors:** Mikito Suzuki, Hirotoshi Horio, Azusa Nakamura, Reiko Shimizu, Toshiyuki Shima, Masahiko Harada, Tsunekazu Hishima

**Affiliations:** 1 Department of Thoracic Surgery, Tokyo Metropolitan Cancer and Infectious Diseases Center Komagome Hospital, 3-18-22 Honkomagome, Bunkyo-ku, Tokyo, 113-8677 Japan; 2 Department of Pathology, Tokyo Metropolitan Cancer and Infectious Diseases Center Komagome Hospital, 3-18-22 Honkomagome, Bunkyo-ku, Tokyo, 113-8677 Japan

**Keywords:** Anterior mediastinal tumor, Computed tomography value, Mediastinal aberrant goiter, Non-enhanced computed tomography, Radiodensity

## Abstract

**Background:**

A mediastinal aberrant goiter is extremely rare, characterized by independence from the thyroid gland. This tumor most commonly develops in the anterior mediastinum and should be differentiated from a thymoma. Moreover, as mediastinal aberrant goiters frequently have a blood supply arising from the thoracic great vessels, preoperative diagnosis and evaluation of the feeding vessel are vital for safe surgery. Herein, we report a rare case of mediastinal aberrant goiter characterized by high radiodensity on non-enhanced computed tomography (CT).

**Case presentation:**

A 77-year-old woman underwent non-enhanced CT, which showed a 2.2-cm, well-circumscribed, homogeneous anterior mediastinal nodule with high radiodensity. The CT attenuation value of the nodule was as high as 91.9 Hounsfield units. A thymoma or mediastinal goiter was suspected owing to the tumor location and the lack of a cystic component. We performed anterior mediastinal tumor resection using video-assisted thoracoscopic surgery. Pathologically, variably sized follicles, indicative of thyroid tissue, were observed with hemorrhage and hemosiderin deposition. Moreover, papillary projections of small follicles (Sanderson’s polsters) were scattered without atypia of the follicular epithelium, indicative of an adenomatous goiter. The diagnosis of a mediastinal aberrant goiter was supported by the absence of an anatomical connection with the thyroid gland. The postoperative course was uneventful, and the patient was discharged on postoperative day 5.

**Conclusion:**

The thyroid gland has a high radiodensity on non-enhanced CT, which correlates with iodine concentration. This radiological feature may be useful for the preoperative differentiation of mediastinal aberrant goiters containing thyroid tissue.

## Background

Intrathoracic goiters are a relatively rare type of goiter, and a mediastinal aberrant goiter without continuity with the thyroid gland is extremely rare [1, 2]. Aberrant mediastinal goiters frequently develop in the anterior mediastinum and should be differentiated from thymomas. Herein, we report a case of a mediastinal aberrant goiter in the anterior mediastinum characterized by high radiodensity on preoperative non-enhanced computed tomography (CT).

## Case presentation

**Fig. 1:**
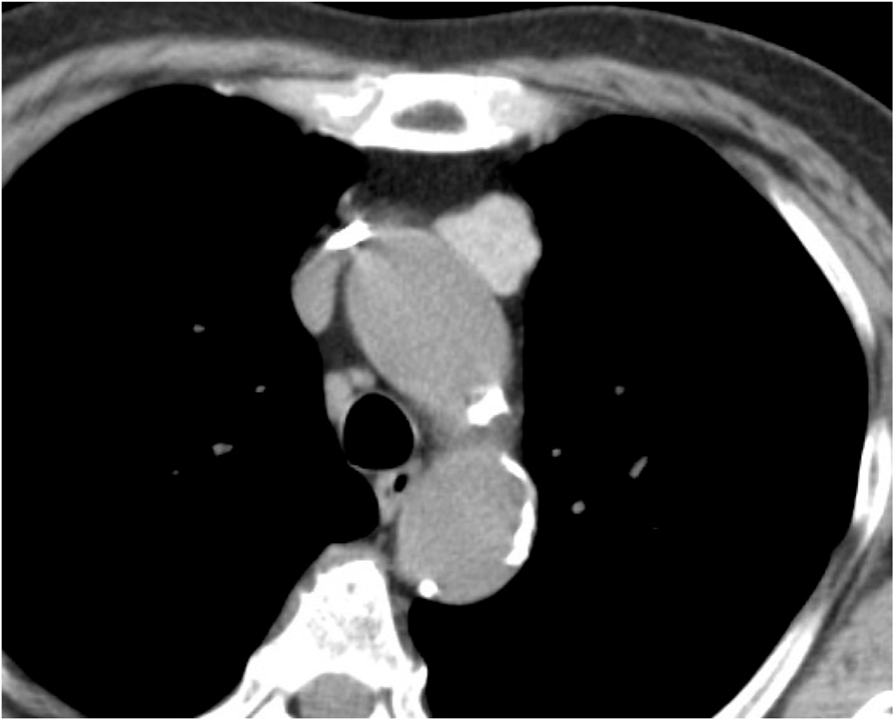
Preoperative imaging findings. Non-enhanced computed tomography (CT) scan. A solitary homogeneous anterior mediastinal tumor (2.0 cm in size) showed high radiodensity (CT value: 91.9 Hounsfield units)

**Fig. 2:**
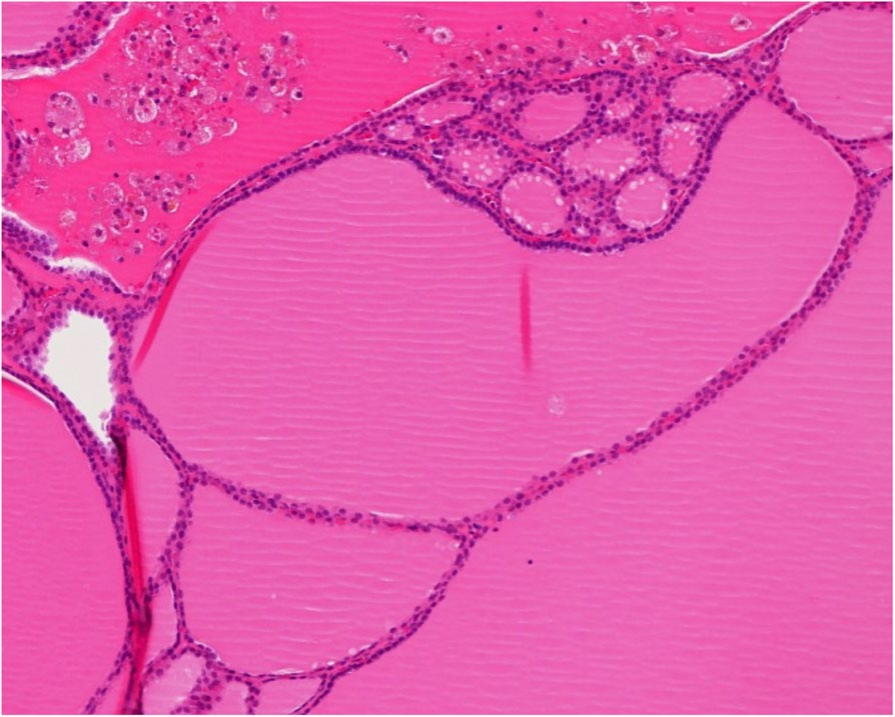
Pathological findings. Unequal-sized thyroid follicles and papillary projections of small follicles (Sanderson’s polsters) were present without atypia of the follicular epithelium

A chest non-enhanced CT scan in a 77-year-old woman with chronic cough revealed a 2.2-cm, well-circumscribed, homogeneous anterior mediastinal nodule with high radiodensity. The CT attenuation value of the nodule was 91.9 Hounsfield units (HU) (Fig. 1). The tumor had no connection to the thyroid gland and did not invade the surrounding organs. The patient’s medical history included second-degree atrioventricular heart block (pacemaker implantation) and diabetes mellitus. Magnetic resonance imaging (MRI) and positron emission tomography (PET)-CT were not performed. As a thymoma or mediastinal goiter was suspected owing to the tumor location and the lack of a cystic component, thoracoscopic anterior mediastinal tumor resection was performed. A lobular tumor was found in the left thymus lobe, but no tumor invasion was detected. The operation time was 1 h 55 min, with 20 g of blood loss. The encapsulated tumor was 2.3 × 2.2 × 1.9 cm in size, with a solid brownish cut surface and scattered hemorrhage deposition. Pathological examination revealed unequal-sized thyroid follicles, Sanderson’s polsters, and papillary projections of small follicles. There was no epithelial atypia and the pathological diagnosis was an adenomatous goiter (Fig. 2). Anatomically, there was no continuity with the thyroid gland, and a mediastinal aberrant goiter was diagnosed. The patient’s postoperative course was uneventful, and she was discharged on postoperative day 5.

## Discussion and conclusions

Rives’ classification is a common anatomical classification system used for intrathoracic goiters [3]. Intrathoracic goiters are classified as substernal or mediastinal aberrant, regardless of whether continuity with the thyroid gland is present. Intrathoracic goiters account for 5% of all mediastinal tumors, and mediastinal aberrant goiters account for 2% of intrathoracic goiters (0.1% of all mediastinal tumors) [1, 2].

Aberrant mediastinal goiters are commonly reported in women ≥50 years. The tumor frequently arises from the anterior superior mediastinum, and blood tests often show euthyroidism [1, 3]. CT scans reveal a well-circumscribed tumor that may be accompanied with focal calcification deposition or cystic degeneration; moreover, enhanced CT shows prolonged enhancement [4, 5]. MRI is reliable for evaluating intrathoracic goiter extension but does not provide additional information in diagnostic yield to that from CT [6]. Significant fluorodeoxyglucose accumulation has been reported on PET-CT [1]. Preoperative differentiation between mediastinal aberrant goiters and thymomas, which is frequently made after excision, has been challenging due to the lack of specific radiological findings on both enhanced CT and MRI. Thyroid scintigraphy is a disease-specific examination for goiters, but limited accumulation has been reported for mediastinal aberrant goiters [5]. The use of preoperative endobronchial ultrasound-guided transbronchial needle aspiration has been reported in cases of intrathoracic goiters located in the superior mediastinum (adjacent to the trachea) [7]. However, a biopsy of intrathoracic goiters in the anterior mediastinum (similar to the goiters in our case) is difficult due to anatomical reasons.

In the thyroid gland, the iodine concentration in thyroid tissue is associated with a high radiodensity on non-enhanced CT [8, 9]. This finding may also apply to mediastinal aberrant goiters comprised of thyroid tissue. However, high radiodensity on non-enhanced CT (91.9 HU) was characteristic of the current case; thus, we suspected a mediastinal aberrant goiter. Mediastinal aberrant goiters have a blood supply arising from the thoracic vessels, such as the aorta or subclavian artery [3]; thus, preoperative diagnosis of mediastinal aberrant goiter allows for evaluation of the feeding vessel, which is necessary for safe surgery, especially in recent years with the increasing use of thoracoscopic and robotic surgery. Although we could not detect obvious blood supply from thoracic great vessels in the present case, an additional preoperative enhanced CT should be performed to preventing unforeseen bleeding.

The median CT value of four intrathoracic goiters on non-enhanced CT in our institution (including two cases of substernal goiter and a mediastinal aberrant goiter in the superior mediastinum) was 76.1 HU (interquartile range: 71.7–89.2). Although there are some reports on mediastinal aberrant goiters, only a few of these have presented the CT values for these goiters. A previous report determined the definite CT values of intrathoracic goiters to be 70–85 HU [9]. This is consistent with our findings. On the other hand, the median CT value of a thymoma was reported to be 39.5 HU (interquartile range: 33.7–42.2) [10], which is lower than that of a mediastinal aberrant goiter. However, intrathoracic goiters with a low concentration of iodine per unit volume, for example, owing to cystic degeneration, may have lower CT values [9]. Although further case accumulation is needed, preoperative non-enhanced CT values may be feasible for differentiating mediastinal aberrant goiters from thymomas.

Although mediastinal aberrant goiters are benign entities, 3–6% of these are known to undergo malignant transformation [11]. Therefore, we believe that surgery should be performed even under preoperative suspicion of a mediastinal aberrant goiter.

We encountered a rare surgical case of a mediastinal aberrant goiter characterized by high radiodensity on non-enhanced CT. An anterior mediastinal tumor with a high radiodensity should be considered a mediastinal aberrant goiter.

## Data Availability

Data sharing is not applicable to this article, as no datasets were generated or analyzed during the current study.

## Abbreviations

CT: Computed tomography
HU: Hounsfield units
MRI: Magnetic resonance imaging
PET: Positron emission tomography
